# Effect of Sustained Administration of Thymol on Its Bioaccessibility and Bioavailability in Rabbits

**DOI:** 10.3390/ani11092595

**Published:** 2021-09-03

**Authors:** Kristina Bacova, Karin Zitterl Eglseer, Gesine Karas Räuber, Lubica Chrastinova, Andrea Laukova, Margareta Takacsova, Monika Pogany Simonova, Iveta Placha

**Affiliations:** 1 Centre of Biosciences, Slovak Academy of Sciences, Institute of Animal Physiology, Soltesovej 4-6, 040 01 Kosice, Slovakia; bacovak@saske.sk (K.B.); laukova@saske.sk (A.L.); takacsova@saske.sk (M.T.); simonova@saske.sk (M.P.S.); 2 University of Veterinary Medicine and Pharmacy, Komenskeho 73, 041 81 Kosice, Slovakia; 3 Institute of Animal Nutrition and Functional Plant Compounds, University of Veterinary Medicine Vienna, Veterinärplatz 1, A-1210 Wien, Austria; karin.zitterl@vetmeduni.ac.at (K.Z.E.); raeuber.gesine@gmail.com (G.K.R.); 4 National Agricultural and Food Centre, Hlohovecka 2, 951 41 Nitra-Lužianky, Slovakia; lubica.chrastinova@nppc.sk

**Keywords:** rabbit, thymol, absorption, distribution, accumulation, excretion

## Abstract

**Simple Summary:**

The purpose of this study was to investigate the bioavailability and metabolic path of thymol, a major constituent of *Thymus vulgaris* L., in the rabbit organism. Oral bioavailability is a key parameter affecting the efficacy of substances, but it is not surprising that it does not correlate satisfactorily with efficacy. The main limitation factors are rate of absorption, metabolism, and excretion processes. In this work, the thymol metabolic path in the rabbit organism was determined for the first time after its sustained oral administration. We confirm intensive absorption of thymol from the gastrointestinal tract; our results point to metabolism and accumulation in kidney tissue and intensive metabolic and excretion processes in the liver. Some metabolic processes were present also after thymol withdrawal. Thymol as a lipophilic substance was found only in trace amounts in fat and muscle tissue as a consequence of its conversion into hydrophilic metabolite and greater elimination in the rabbit organism. This paper highlights the insufficient knowledge of modes of action of plant compounds in animal organisms.

**Abstract:**

The objective of this study was the detection of thymol in rabbit plasma, tissues, large intestinal content, and faeces. Forty-eight rabbits were divided into control and experimental groups (thymol 250 mg/kg feed). Thymol was administered for 21 days and then withdrawn for 7 days. Concentration of thymol in the intestinal wall (IW) was significantly higher than in plasma (*p* < 0.05) and liver (*p* < 0.05); in the kidneys it was significantly higher than in plasma (*p* < 0.05) and liver (*p* < 0.05) during thymol addition. Thymol in IW was significantly higher than in plasma also after withdrawal (*p* < 0.01). Significant correlation (r_s_ = −1.000, *p* < 0.01) between IW and plasma points to the intensive absorption of thymol from the intestine, while the correlation between plasma and liver (r_s_ = 0.786, *p* < 0.05) indicates intensive biotransformation and excretion processes in liver. Significant correlation between liver and kidney (r_s_ = 0.738, *p* < 0.05) confirms the intensive metabolism of thymol in the kidney. During the withdrawal period, thymol was detected above trace amounts only in faeces, and was significantly higher than in the colon during both periods (*p* < 0.01). Results show intensive biotransformation of thymol in the rabbit organism.

## 1. Introduction

In recent years, natural products have assumed great importance as antibiotic replacement additives and as growth promoting agents in food animals. There is large pressure on the animal production industry to improve animal treatment as well as production performance, and to ensure the safety of products for human consumption while minimizing economic losses [1]. Even though, the benefits of herbal additives depend on the biological activities of their compounds and their pharmacokinetics, their precise mode of action at the molecular level has not yet been fully elucidated [2,3].

To our knowledge, bioaccessibility, bioavailability, and metabolism of phenolic compounds have been studied in vitro in humans [4,5] and in chickens [6]. No information is available about absorption, distribution, and deposition of natural compounds at target sites in the rabbit organism. The rabbit gastrointestinal tract has characteristic features compared with other animal species, such as the relative importance of the well-developed caecum, and a separation mechanism within the proximal colon [7]. The efficiency of the rabbit’s digestion depends in large part on the production and ingestion of caecotrophes, which must be considered as an integral part of the rabbit´s digestion system. The process of caecotrophy may be considered as “pseudorumination” which improves feed utilization [8,9].

One of the crucial aspects of the beneficial effect of natural compounds is the amount present in the gut as a result of their release from feed, and their consequent ability to pass through the intestinal barrier. Intestinal absorption of many compounds is limited by a range of biological and physiological barriers in the gastrointestinal tract. Biological barriers are represented mainly by the mucus layer and epithelial cell layer, which is composed of villus and crypt cells. Physiological factors include enzymatic activities in the intestinal lumen, specific transport mechanisms which are able to limit absorption, and intestinal transit time [10].

In terms of the potential role of thymol as feed additive for animals, the aim of our study was to try to produce a more detailed view and better understanding of the mechanism of its absorption, distribution, and accumulation in the rabbit organism after its sustained application into the rabbit’s diet.

## 2. Materials and Methods

### 2.1. Animals Care and Use

The trial was carried out at the experimental rabbit facility of the National Agricultural and Food Centre, Research Institute for Animal Production, Nitra, Slovakia. The protocol was approved by the Institutional Ethical Committee, and the State Veterinary and Food Office of the Slovak Republic approved the experimental protocol (4047/16-221).

### 2.2. Animals and Housing

After weaning at 35 days of age, 48 rabbits of both sexes (meat line M9) were randomly divided into a control group (CG) fed a standard diet and an experimental group (EG) fed a standard diet into which 250 mg/kg of thymol was incorporated in powder form (≥99.9%, Sigma-Aldrich, St. Louis, USA). All experimental wire-net cages (61 cm × 34 cm × 33 cm) were kept in rooms with automatic temperature control (22 ± 4 °C) and photoperiod (16L:8D). The rabbits could feed ad libitum and had free access to drinking water. The experiment lasted 28 days. The rabbits received feed with thymol addition for 21 days (56 d of age) and for the next 7 days (63 d of age) the thymol was withdrawn. Initial live weight was 1006 ± 98 g in CG and 1035 ± 107 g in EG (2044 ± 24 g in CG, 1965 ± 58.7 g in EG at 56 d of age, and 2671 ± 72 g in CG, 2796 ± 60 g in EG at 63 d of age). Eight rabbits in each group were killed at 56 or 63 d of age using electronarcosis (50 Hz, 0.3A/rabbit for 5 s), immediately hung by the hind legs on the processing line and quickly bled by cutting the jugular veins and the carotid arteries.

### 2.3. Diet and Chemical Analyses

The standard diet consisted of a commercial diet for growing rabbits (KKZK, Liaharensky podnik Nitra a.s., Nitra, Slovakia) with ingredients and chemical composition as shown in Table 1. The diet was administered in the form of pellets with an average size of 3.5 mm. The feed was stored in darkness to protect against degradation processes. The Association of Official Analytical Chemists (AOAC) methods [11] were used to determine the proportions of crude protein (no. 990.03, CP), ash (no. 942.05), and dry matter (no. 967.03, DM) in the diet, while DM amount was also determined for the tissues, gut content, and faeces. Neutral detergent fibre (NDF) and acid detergent fibre (ADF) were analysed according to Van Soest et al. [12].

### 2.4. Thymol Stability in Feed

Thymol evaporation in feed was analysed every week during thymol application using high-performance liquid chromatography (HPLC) according to the modified method of Oceľová [6] and Pisarčíková et al. [13]. Samples were analysed in triplicate. Briefly, 2 mL of methanol was added into a glass tube containing 0.2 g of milled feed and thymol was extracted in an ultrasonic bath. The methanolic extract was then analysed using the HPLC method with an Ultimate 3000 HPLC-system liquid chromatograph (Dionex, Sunnyvale, CA, USA). The chromatographic analyses were evaluated by means of Chromeleon^®^ Software Version 6.80 SR10 Build 2906 (Thermo Fisher Scientific, Waltham, MA, USA).

### 2.5. Sampling

To determine the thymol content in plasma, blood (1.5 mL) from eight rabbits was collected from the marginal ear vein (*vena auricularis*) into heparinized Eppendorf tubes and plasma was obtained after centrifugation at 1180× *g* for 15 min. The gastrointestinal tract was removed from the body cavity and was divided into small intestine, caecum, and colon (n = 8). Caecum and colon content were removed, and the small intestinal lumen was gently washed with 0.9% NaCl solution. Obtained samples of gut content and intestinal wall together with plasma, liver, kidney, muscle (*musculus longissimus dorsi*) and spleen tissue, fat, and faeces were immediately frozen in liquid nitrogen and stored at −70 °C until analysis. All samples were collected at both experimental days (56 or 63 d of age).

### 2.6. Thymol Analyses in Plasma, Tissues, Large Intestinal Content and Faeces

Detection of thymol in samples of plasma, tissues and faeces was performed using headspace solid-phase microextraction followed by gas chromatography coupled with the mass spectrometry method as described by Bacova et al. [14] and Placha et al. [15]. Briefly, detection and quantification were carried out using a GC/MS (type HP 6890 GC) system coupled with a 5972-quadrupole mass-selective detector (Agilent Technologies GmbH, Wilmington, DE, USA). Detection of thymol was confirmed by comparing its specific mass spectrum and retention time with those of the reference compound. Additionally, the Kovats index was calculated. Calibration curves were generated by plotting the peak-area ratios of thymol to o-cresol used as an internal standard (Sigma-Aldrich, St Louis, MO, USA) against the known thymol concentrations. The selective ion mode was used for quantitative analysis of thymol. The mass fragments m/z 135 and m/z 150, as well as m/z 107 and m/z 108, were monitored as characteristic for thymol and o-cresol, respectively. Calibration curves were prepared from blank samples spiked directly with 50 µL thymol (Applichem, Darmstadt, Germany) in standard solutions with known concentrations as follows: for plasma 48, 100, 200, 400, and 800 ng of thymol per mL, for intestinal wall 100, 200, 400, 800, 1000 ng; for liver, kidney, muscle, caecum and colon content 100, 200, 400, 800, 1000, 2000 ng; spleen, fat 24, 50, 100, 200, 400 ng; faeces 200, 400, 800, 1600, 2000, 4000 ng of thymol per g of tissue. Each point on the calibration curve was analysed as a duplicate. The peak of thymol was detected around 19 min and the o-cresol peak occurred around 10 min in all samples. Samples for thymol detection were prepared using the method described by Oceľová et al. [16]. Enzyme β–Glucuronidase Helix pomatia Type HP-2 (aqueous solution, ≥100,000 units/mL, Sigma-Aldrich, St Louis, MO, USA) was added to samples to cleave thymol from its glucuronide and sulphate, since only free thymol should be detected in the GC system.

### 2.7. Statistical Analysis

Data collected were analysed using the Kolmogorov–Smirnov test for normal and non-normal distribution. All data were not accepted as parametric. The Kruskal–Wallis test with post hoc Dunn´s Multiple Comparison test was used to determine the differences between plasma and tissues or caecal, colon content and faeces. Results are presented as mean value ± standard error of mean (SEM). Differences were considered significant at *p* < 0.05. Correlations of thymol concentrations between plasma and intestinal wall, plasma and liver, and liver and kidney were analysed using nonparametric Spearman’s Rank Correlation and expressed as Spearman’s correlation coefficient (r_s_). Statistical analyses were performed using Graph Pad Prism (GraphPad Software, San Diego, CA, USA). The experimental unit was the animal’s cage.

## 3. Results

### 3.1. Thymol Stability in Feed

Concentration of thymol in feed during the period of the experiment with its addition was relatively stable at 274 µg/g DM–0 d; 255 µg/g DM–7 d; 236 µg/g DM–14 d.

### 3.2. Thymol in Plasma and Tissues

**Figure 1:**
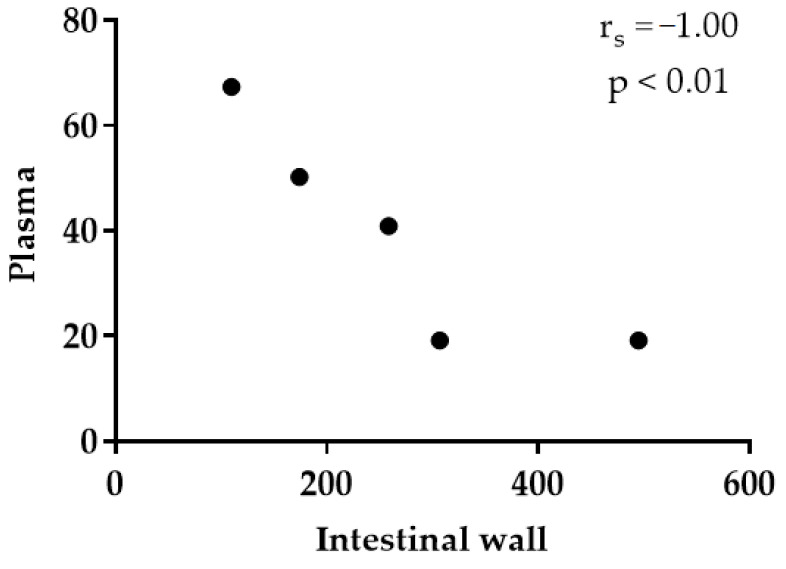
Correlation between plasma (ng/mL) and intestinal wall (ng/g DM).

**Figure 2:**
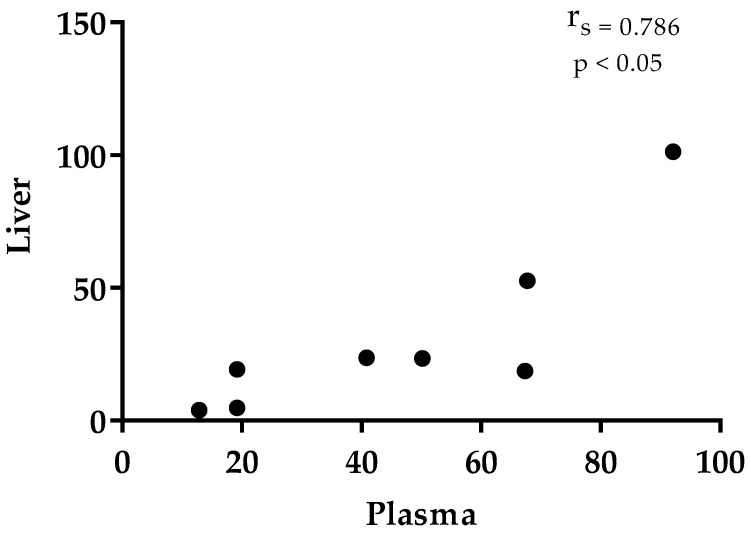
Correlation between liver (ng/g DM) and plasma (ng/mL).

**Figure 3:**
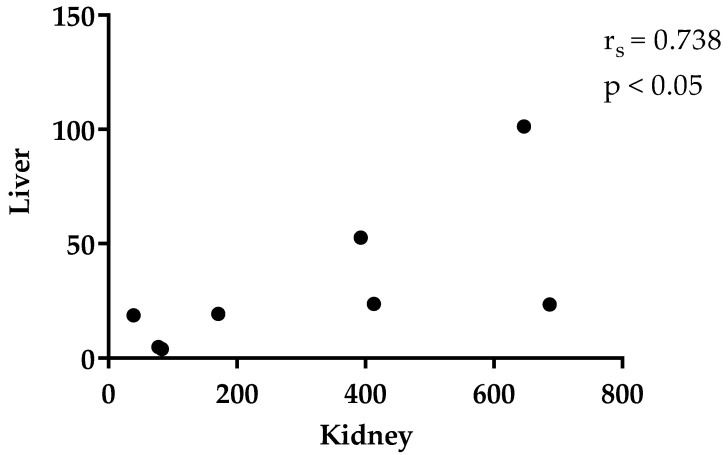
Correlation between liver and kidney (ng/g DM).

Level of thymol in the intestinal wall was significantly higher than in plasma (*p* = 0.0211) and liver (*p* = 0.0305), and in the kidneys it was significantly higher than in plasma (*p* = 0.0259) and liver (*p* = 0.0415) during the period of thymol addition (Table 2). Thymol in fat (19.9 ± 7.36 ng/g DM, n = 2) and muscle (26.6 ng/g DM, n=1) during this period was found only in a small number of samples, and only in trace amounts in others. For this reason, those samples were not included in the statistical evaluation. Significant correlation was established between thymol concentration in plasma and intestinal wall (r_s_ = −1.0, *p* < 0.01), plasma and liver (r_s_ = 0.786, *p* < 0.05) and liver and kidney (r_s_ = 0.738, *p* < 0.05, Figure 1, Figure 2 and Figure 3). Even though thymol content was determined only in trace amounts during the period without thymol addition, the differences were statistically evaluated. Thymol in intestinal wall was significantly higher than in plasma in this period (*p* = 0.0035, Table 2).

### 3.3. Thymol in Caecum, Colon and Feces

Thymol in faeces was significantly higher than in the colon during both experimental periods, even if only in trace amounts without further thymol addition (*p* < 0.01, Table 3). During this period only thymol in faeces was detected above trace amount.

## 4. Discussion

The epithelial cells in the small intestine wall contain various metabolic enzymes and transporters. The intestinal microflora possesses a wide range of metabolic processes including hydrolysis of glucuronides, sulphate esters, and amides. Enzymes in the intestinal microflora can hydrolyse drug metabolites, especially glucuronide conjugates, and convert them back to the parent compound [10]. The parent drugs excreted and/or released by the action of gut microflora are reabsorbed by intestinal cells. Metabolites are continuously excreted into the large intestine, where they are again hydrolysed and reabsorbed [17].

After being absorbed from the GIT, thymol becomes metabolized during processes of biotransformation and becomes more hydrophilic. The metabolites, mainly sulphates and glucuronides, are transported across the intestinal epithelium by active processes involving transmembrane proteins. Many transporters such as peptides, vitamins, amino acids, and sugars play important roles in the translocation of drugs and were identified in large amounts in the GIT [18].

Rubió et al. [19] and Pisarčíková et al. [13] have confirmed that thymol, which is a small lipophilic molecule, is not detected in unmetabolized form in plasma, as they detected only its conjugates (thymol sulphate and glucuronide). They also detected thymol conjugates in the duodenal wall, which points to active biotransformation of thymol in the organism.

Significant correlation (r_s_ = −1.00, *p* < 0.01) between thymol content in the intestinal wall and plasma in our experiment indicates intensive absorption of thymol from the intestine. Placha et al. [15] and Oceľová et al. [20] also confirmed the intensive absorption of thymol from all intestinal segments in broiler chickens after four weeks of diet supplementation with thyme essential oil. They found significant correlation between thymol content in plasma and individual intestinal segments. Although we found six times lower concentration of thymol in plasma during its addition and seven times lower after its withdrawal (even if only in trace amounts) in comparison with the intestinal wall, we can confirm that some metabolic processes were still active after thymol withdrawal from feed (Table 2 and Table 3).

The rabbit caecum is the largest part of the large intestine and contains approximately 40% of the intestinal content. The primary mechanism by which nutrients are released from intestinal content is microbial fermentation, and its products are absorbed through the intestinal wall or are reingested as caecotrophes. The retained particles from the proximal part of the colon also provide substrate for caecal microbiota. The mucus which coats the caecotrophes protects them and allows the fermentation processes to continue in them until they reach the intestine. The composition of the intestinal flora depends to a large extent on the caecotrophic microbial population [21].

Intestinal microflora and epithelial cells play a crucial role in metabolic processes because they produce a wide range of metabolic enzymes [22]. The counts of bacterial flora are highly variable in different parts of the gastrointestinal tract [23]. We assume that the metabolic enzymes responsible for thymol biotransformation in the caecum and consequently in caecotrophes are expressed in large enough amounts that they can affect metabolic activities and consequently exert influence on the amount of thymol and its metabolites in the caecum.

There are some conditions affecting the absorption of compounds in the GIT. In addition to their rate of dissolution in the intestinal fluids, they must also be able to cross membranes in each part of the GIT. In case they are not able to cross these membranes by the time they reach the colon, the extent of intestinal absorption is not sufficient, and the compounds are excreted in faeces [24]. All these circumstances may explain the high concentrations of thymol in the caecum and faeces, not only during its addition but also after its withdrawal from feed (Table 3).

The first study which confirmed the presence of thymol metabolites (thymol sulphate and glucuronide) in the duodenal wall of broiler chickens after sustained consumption of thyme essential oil also confirmed the key role of the intestine in the metabolism of thymol [13]. Oceľová [6] detected thymol in the liver at a level of 8.9% of its concentration in the intestinal wall and observed significant correlation between thymol concentrations in liver and plasma, and liver and intestinal wall, which might indicate sufficient absorption of thymol from the intestinal wall to the liver through the vena portae. We found 15% (with thymol) and 29% (without thymol) of thymol in liver compared with its content in the intestinal wall, and significant correlation between thymol concentrations in plasma and liver (r_s_ = 0.7857, *p* < 0.05). These results are in agreement with the findings of the above-mentioned authors and point to intensive biotransformation and excretion processes in the liver.

Between the enzyme systems and efflux transporters there exist mutual processes which can affect the efficiency of drugs in the intestine. These couplings can prolong the exposure of drugs in vivo and are crucial for enteric and enterohepatic recycling. Some parent compounds are absorbed and metabolized in the intestinal cells, while some portions are effluxed back into the intestinal lumen or transported to the mesenteric vein and are taken up by hepatocytes [17]. Excreted metabolites from enterocytes are converted back to the parent compound and again reabsorbed. Compounds in the liver are metabolized and together with parent compounds are excreted with bile into the duodenum and are then reabsorbed there [14,17]. These repeating processes together with processes of caecotrophy could explain the thymol content in plasma, tissues, and intestinal content after thymol withdrawal, although only in trace amounts. We must point out that we detected the sum of metabolized and non-metabolized thymol, as the thymol metabolites were cleaved by added enzyme, β-glucuronidase. However, the extent of absorption of compounds (drugs) and the contribution to their bioavailability by metabolic enzymes and transporters or receptors present on the intestinal membrane are still not clear.

In the present study, a significantly higher level of thymol in the kidney in comparison with liver tissue and plasma confirmed its metabolism and/or accumulation in this organ (Table 2). Our results are in agreement with our previous study [6], in which we detected a significantly higher thymol concentration in the kidney of chickens with thyme essential oil diet supplementation. Takada [25] detected thymol glucuronide and thymol sulphate in the urine of rabbits, and to our knowledge this is the only study concerned with the metabolic path of thymol in these animals. Data available so far indicate that thymol sulphate could be reabsorbed in the proximal tubule after glomerulary filtration, and that cleavage of this metabolite is achieved by enzymes located at the luminal brush border, so that subsequently thymol could be reabsorbed [26]. These authors demonstrated that although the liver is the most important organ for biotransformation, kidney microsomes demonstrate more effective metabolic processes than liver or intestinal microsomes. The significantly higher content of thymol in the kidney and the correlation between liver and kidney (r_s_ = 0.7381, *p* < 0.05) in our study also confirm the intensive metabolism in kidney tissue. However, further study of renal metabolism mechanisms is necessary to confirm these findings.

Before drug compounds can reach systemic circulation and therapeutic targets, several barriers must be overcome [18]. Although lipophilic molecules evidently pass the barriers with ease by transcellular routes, various efflux transporters are preferentially bound with lipophilic molecules, seriously limiting their absorption. The first-pass metabolism which occurs in the intestinal wall decreases the chance of molecules which get into epithelial cells to reach the systemic circulation. Once compounds are absorbed in the GIT, the next major barrier awaits in the form of the first-pass metabolism in the liver. Drugs passing through the portal vein encounter hepatocytes and are metabolized there [18]. Based on these processes we can explain the trace amounts of thymol detected in rabbit muscle and fat in our experiment. Trace amounts of thymol in the muscle tissue of broiler chickens were also detected by Haselmeyer et al. [27] after thymol addition (55 mg/kg DM feed), and by Oceľová [6] after thyme essential oil application. Lipophilic substances which are not metabolized during the process of biotransformation are less readily eliminated and accumulate in fat tissue [22]. As we found only traces of thymol in this tissue, we can hypothesize that the majority of thymol was metabolized and converted into hydrophilic substances.

Finally, little is known about the bioactivity of thymol and thymol metabolites, further studies are needed to evaluate the distribution of thymol in different tissues at different levels and establish its suitable concentration for a beneficial effect on animal health.

## 5. Conclusions

Our results showed that, thymol was efficiently absorbed from the intestinal lumen and intensive metabolic processes in liver and kidney were observed, while accumulation in fat and muscle tissue was low, probably due to its intensive biotransformation into hydrophilic substances which were then excreted. We confirm some metabolic processes involving thymol even after its withdrawal from feed, as a consequence of caecotrophy. Oral bioavailability of plant compounds is a challenge for scientists because their metabolic processes in the animal organism should be understood at molecular level. 

## Figures and Tables

**Table 1:** Ingredients (%) and chemical composition (g/kg feed) of experimental diet.

Ingredients (%)		Chemical Composition (g/kg Feed)	
Dehydrated lucerne meal	36.0	Dry matter (g/kg)	900.9
Dry malting sprouts	15.0	Organic compounds	831.8
Oats	13.0	Nitrogen free extract	444.3
Wheat bran	9.0	Neutral detergent fibre (NDF)	352.9
Barley	8.0	Acid detergent fibre (ADF)	208.1
Extracted sunflower meal	5.5	Crude fibre	177.8
Extracted rapeseed meal	5.5	Crude protein	176.6
Dried distiller grains with solubles	5.0	Cellulose	163.1
Premix ^1^	1.7	Hemicellulose	144.8
Limestone	1.0	Starch	133.1
Sodium chloride	0.3	Ash	69.2
		Fat	33.1
		Metabolic energy, MJ/kg	9.9

^1^ The vitamin-mineral premix provided per kg of complete diet: Retinyl acetate 5.16 mg, Cholecalciferol 0.03 mg, Tocopherol 0.03 mg, Thiamin 0.8 mg, Riboflavin 3.0 mg, Pyridoxin 2.0 mg, Cyanocobalamin 0.02 mg, Niacin 38 mg, Folic acid 0.6 mg, Calcium 1.8 mg, Iron 70 mg, Zinc 66 mg, Copper 15, Selenium 0.25 mg.

**Table 2:** Thymol content in plasma (ng/mL) and tissue (ng/g DM).

	56 d of Age (with Thymol)		63 d of Age (without Thymol)	
	Mean	SEM	Mean	SEM
Plasma	46.2 ^b^	10.0	2.73 ^b^	0.458
Intestinal wall	268 ^a^	65.9	20.4 ^a^	2.70
Liver	39.9 ^b^	13.4	5.93 ^ab^	0.285
Kidney	314 ^a^	91.7	16.1 ^ab^	6.53
Spleen	181 ^ab^	40.1	ND	-

^a,b^ Values within a column with different superscript letters differ significantly (*p* < 0.05). Data are presented as mean ± standard error of mean (SEM).

**Table 3:** Thymol content in caecum, colon, and faeces (ng/g DM).

	56 d of Age (with Thymol)		63 d of Age (without Thymol)	
	Mean	SEM	Mean	SEM
Caecum	882 ^ab^	231	45.8 ^ab^	12.44
Colon	672^b^	330	16.4 ^b^	9.44
Faeces	2444 ^a^	451	150 ^a^	40.54

^a,b^ Values within a column with different superscript letters differ significantly (*p* < 0.05). Data are presented as mean ± standard error of mean (SEM).

## Data Availability

Data availability upon reasonable request to the corresponding author.
